# Determinants of financial poverty alleviation efficiency: Evidence from Henan, China

**DOI:** 10.1371/journal.pone.0277354

**Published:** 2022-11-10

**Authors:** Qitao Liu

**Affiliations:** School of International Education, Xuchang University, Xuchang, Henan, China; Universiti Malaysia Sabah, MALAYSIA

## Abstract

Poverty alleviation is a common cause for all human beings. The purpose of this study is to evaluate the efficiency of financial poverty alleviation in 18 cities in Henan, China, and to explore the factors affecting the efficiency of financial poverty alleviation, so as to contribute new knowledge to the cause of poverty alleviation. Based on the relevant data of 18 cities in Henan, using output-oriented DEA model and Tobit regression model with bootstrap method, this study evaluates the efficiency of financial poverty alleviation in various cities in Henan, and explores the determinants of the efficiency of financial poverty alleviation. The results show that the overall poverty alleviation efficiency of Henan is high, and the financial poverty alleviation efficiencies of different cities show distinct heterogeneities. The efficiencies of financial poverty alleviation in Zhengzhou and Luoyang are 1, and there are different spaces for improvement in the efficiency of financial poverty alleviation in other cities. Financial subsidies are the most important positive factors affecting the efficiency of financial poverty alleviation. For every 1% increase in the value of financial subsidies, the poverty alleviation efficiency will increase by 0.213%. The urban-rural dualistic economic structure is negatively correlated with the efficiency of financial poverty alleviation. Every 1% increase in the value of the urban-rural dualistic economic structure will reduce the poverty alleviation efficiency by 0.11%. Industrial structure is positively related to the efficiency of financial poverty alleviation. For every 1% increase in the value of the industrial structure, the poverty alleviation efficiency will increase by 0.072%. The formulation of financial poverty alleviation policies in various regions should be combined with their own characteristics, and promote the efficiency of financial poverty alleviation by strengthening the advantages and making up for the deficiencies.

## Introduction

Eradicating poverty is the biggest global challenge facing the world today [1, 2]. In the process of global governance, the inevitable problem is poverty eradication [3]. As a worldwide problem, anti-poverty has always been an arduous task for mankind. In 2000, the first goal of the Millennium Development Goals adopted by the United Nations was to eliminate more than half of the world’s poor people by 2015. In the 2030 agenda for sustainable development adopted at the summit of the 193 member states of the United Nations, "eradicating poverty in all forms in the world" ranked first among the 17 Sustainable Development Goals [4]. Although the global poverty problem has been improved in development, it cannot be ignored that a large number of people are still stuck in poverty. According to the 2020 Global Multidimensional Poverty Index (MPI) released by United Nations Development Programme (UNDP), 1.3 billion people (22%) in 107 developing countries lived in multidimensional poverty. Before the outbreak of COVID-19 pandemic, global poverty was falling every year. However, according to the report released by the World Bank in January 2021, the epidemic had made more than 700 million people in the world face extreme poverty, which was the first increase in global poverty in the past 20 years and being likely to continue. Although no data are available to measure the increase in global poverty following the pandemic, simulations based on different scenarios in 70 developing countries suggest that the world’s progress in tackling multidimensional poverty could be set back by 8~10 years if the pandemic is not addressed. While significant progress has been made in global poverty reduction, the world still faces enormous challenges in order to achieve the United Nations Sustainable Development Goals of eradicating poverty in all its forms by 2030.

China’s poverty alleviation and development is an important part of the global poverty reduction cause, and has received long-term support from the UNDP and other international agencies. The Chinese government has incorporated poverty alleviation and development into the strategic layout of "four comprehensives" and vigorously implemented targeted poverty alleviation. On November 23, 2020, Guizhou announced that the last nine deep poverty-stricken counties would be withdrawn from the poverty-stricken county series, which not only marked the overall poverty eradication of 66 poverty-stricken counties in Guizhou, but also marked the removal of all 832 poverty-stricken counties designated by the Chinese government and the completion of the national poverty eradication goal. In June 2018, an exhibition on China’s achievements in poverty alleviation was held at the UN headquarters under the theme of "Pursuing a Better Life". It is the first time that China has held an exhibition on poverty eradication on the multilateral stage. Ms. Amina Mohammed, the Deputy Secretary General of the UN, believed that over the past few decades, China had made great achievements in poverty reduction and contributed greatly to all mankind. About 800 million people have been lifted out of poverty according to the international poverty line standards [5]. China’s success in poverty eradication provides multiple lessons for other developing countries in implementing the UN 2030 Agenda for sustainable development, and encourages other countries to achieve the UN Sustainable Development Goals [6].

There are many ways of poverty alleviation, including industrial poverty alleviation [7], education poverty alleviation [8], health poverty alleviation [9], photovoltaic poverty alleviation [10], e-commerce poverty alleviation [11, 12], etc. Financial poverty alleviation refers to the use of bank credit funds or cooperation with domestic and foreign financial institutions to engage in industrial development and improve the production and living conditions of poor areas and poor farmers [13]. Among the various successful poverty alleviation models, financial poverty alleviation is characterized by abundant resources, market-oriented operation, strong sustainability of poverty alleviation effect, etc. At the same time, there are also problems such as single subject, lack of products, inaccurate investment direction, and unsatisfactory poverty alleviation efficiency. Measuring the efficiency of financial poverty alleviation, comparing regional differences [14], and analyzing the determinants affecting the efficiency of financial poverty alleviation are not only conducive to the construction of long-term mechanism of financial poverty alleviation, but also can optimize regional coordination strategies to achieve the sustainable goal of financial poverty alleviation.

Because of the heterogeneities of endowments and development levels, there are differences in the efficiency of financial poverty alleviation among regions. Ignoring these differences may have a negative impact on the sustainable development of poverty alleviation and the healthy development of finance. To fill this gap, based on the basic assumption of variable return to scale, this study uses DEA-BCC model to calculate the efficiency of financial poverty alleviation of cities in Henan, and explores the root causes of the differences of the efficiency of financial poverty alleviation, which has important theoretical and practical significances for optimizing the allocation of resources, consolidating the achievements of poverty alleviation, and orderly linking rural revitalization.

### Literature review

In recent years, scholars around the world, including China, have conducted extensive research on financial poverty alleviation. Representative achievements mainly include the role, path and mode of financial development in poverty alleviation, and the difficulties, dilemmas and performance of financial poverty alleviation and other areas.

Firstly, financial development helps alleviate poverty [15], and the role of financial poverty alleviation is both direct and indirect. Existing studies [16–19] generally believe that financial development reduces poverty mainly through direct means such as raising the income level of the poor, reducing inequality in income distribution, and expanding the coverage of financial services, while alleviating poverty indirectly by promoting capital accumulation, boosting technological innovation, and influencing economic growth. Levine [20] found that financial development can significantly reduce income inequality (Gini coefficient) and increase the average income level of the majority (about 80%) of the lower-income population by eliminating the credit constraints of the poor. King and Levine [21] found that there was an obvious positive correlation between national financial development and economic growth. Cristian [22] found that financial development can achieve poverty alleviation by promoting economic growth. Inclusive Finance helped the poor obtain credit resources and realize asset accumulation [23]. Boukhatem [24] found that financial development had a direct, non-linear effect on poverty reduction. Bayar et al. [25] used panel unit root test and cointegration test to empirically analyze the relationship between financial development and poverty reduction in emerging market economies from 1993 to 2012, and the results showed that financial development, including banking industry and stock market development, had a significant positive impact on poverty reduction. Zahonogo [26] built a poverty model and made an empirical analysis of how financial development affecting poverty indicators. It was found that the relationship between financial development and poverty reduction was nonlinear, and there was a threshold effect. Yang [27], Chen and Shi [28] believed that the improvement of the degree of rural financialization and its efficiency can promote the rural poverty alleviation. However, some scholars [29, 30] believed that the development of Microcredit and Inclusive Finance cannot play a positive role in poverty alleviation for all poor people because extremely poor people prefer to get employment opportunities and social security. Using Nigeria’s 1996–2017 data and ARDL method, the research of Olohunlana and Dauda [31] showed that the impact of financial development on poverty reduction was negligible. Kaidi et al. [32] studied the relationship between financial development and poverty reduction using panel data from 1980 to 2014 in 132 countries around the world and the three-stage least square method. The results showed that financial development could not improve the situation of the poor. Moreover, financial fluctuations [33, 34] and excessive income distribution gap [30, 35] would all have adverse effects on poverty alleviation. It can be found that there is no consensus on the role and efficiency of finance in poverty alleviation.

Secondly, the efficiency of financial poverty alleviation is a hot research topic in academia. The fundamental problem of management is efficiency [36]. Improving the efficiency of financial poverty alleviation means empowering poor areas or poor people, helping them to rejuvenate and find the path to prosperity. To improve efficiency, the first mission is to measure efficiency whose priority is to construct a system of evaluation indicators. Tang et al. [37] took 30 provinces in China from 2013 to 2016 as the research object, and employed DEA method to estimate the poverty alleviation efficiency of E-commerce with the gross domestic product per capita as the output index and the number of township cultural stations as the input index; employed the intermediary effect model to analyze the influence of intermediary variables, fiscal revenue and human capital, on the poverty alleviation efficiency of E-commerce. However, this study lacks attention to micro-regions. Appiahtoo and song [38] used the inclusive finance index to study the poverty alleviation efficiency of fintech and its impact on China’s poverty reduction, but did not pay attention to the huge poverty heterogeneity among China’s provincial regions. In addition, China’s current difficulties in financial poverty alleviation are mainly the imperfect financial supervision system [39], the high cost of financial poverty alleviation [40], the low enthusiasm of financial institutions for poverty alleviation [41, 42], the difficulty of financial development to meet the increasing demand for funds [43], the lagging rural insurance system [44], the high risk of poverty alleviation credit [45], and the poor poverty reduction effect of financial poverty alleviation funds [46].

Thirdly, the performance of financial poverty alleviation in China varies depending on the poverty alleviation method, subjects and regions, and the sustainability of financial poverty alleviation faces challenges. A study by Tu [47] using DEA model showed that there were discrepancies in the efficiency of financial poverty alleviation among different regions, and the two main influencing factors were loan investment structure and social credit environment. Based on the stochastic frontier analysis method, Li and Feng [48] concluded that there was still more space to improve the efficiency of financial poverty alleviation, and the efficiency of industrial poverty alleviation loans to drive poor households was higher than the efficiency of poverty alleviation microcredit. Wang et al. [49] argued that there were large differences between the efficiency of financial poverty alleviation in different regions, and it would be affected by the differences in external environment and random errors. Li and Han [50] claimed that the role of formal finance and financial poverty alleviation funds on rural poverty alleviation was not significant, and the overall level of rural financial poverty alleviation efficiency was not high and showed a spatially unbalanced distribution of high in the south and low in the north, high in the east and low in the west. Ji [51] contended that the sustainability of financial poverty alleviation was seriously affected by factors such as insufficient supply of funds and imperfect supporting policies.

Reviewing of the above research findings reveals that various new advances have been made on financial poverty alleviation in the past two or three decades [52, 53], but there are still numerous shortcomings and no definite conclusion has been reached so far. First, There are still few literatures that directly study the performance of financial poverty alleviation, especially for a certain region. The efficiency of poverty alleviation has significant heterogeneity among regions, and in addition to a large number of macro-level analyses, a large number of micro-level studies are needed to fill the gap. Second, the literature on empirical studies of financial poverty alleviation efficiency at the micro level in existing studies is unsystematic, and there are rare comprehensive analyses of the influencing factors of financial poverty alleviation efficiency, which is the key to put forward practical suggestions for optimizing countermeasures for financial poverty alleviation efficiency. The shortcomings of the existing research mentioned above raises the necessity of new evidence. This study takes the financial poverty alleviation practices of 18 cities in Henan as the research object, evaluates the financial poverty alleviation performance with DEA model, analyzes the determinants of the efficiency of financial poverty alleviation with Tobit model. In addition, this study analyzes the determinants from three aspects: the highly typical dualistic economic structure of China, the highly typical industrial structure of Henan, and financial subsidies,which is one of the main determinants of financial poverty alleviation. At last, this study also proposes policy recommendations to optimize the efficiency of financial poverty alleviation, with a view to contributing to the practice of financial poverty alleviation worldwide.

### Data source and methods

#### Study area

Henan is a provincial administrative region of China with Zhengzhou as the capital, and is located in the heart part of China. Henan is bounded by 31°23’-36°22’ north latitude and 110° 21’-116°39’ east longitude. It lies in the mid-lower reach of the Yellow River, with an area of 160 thousand square kilometers, and a population of nearly 98.83 million by the end of 2021. Henan is a traditional agricultural province and populous province, but also a growing economy and industry province. Henan is an important comprehensive transportation hub and the center of the flow of people and logistics in China.The terrain is high in the west and low in the east, which consists of plains and basins, mountains, hills and water surfaces. Most regions are located in the warm temperate zone, and the south is trans-subtropical, which belongs to the continental monsoon climate of transition from north subtropical zone to warm temperate zone. Located at the junction of the open coastal areas and the backward western areas, it is the middle zone of China’s economic development from east to west. Henan has 18 prefecture-level cities. The regional GDP of Henan is 5,888.741 billion yuan, and the three industrial structures are 10:41:49.

**Fig 1:**
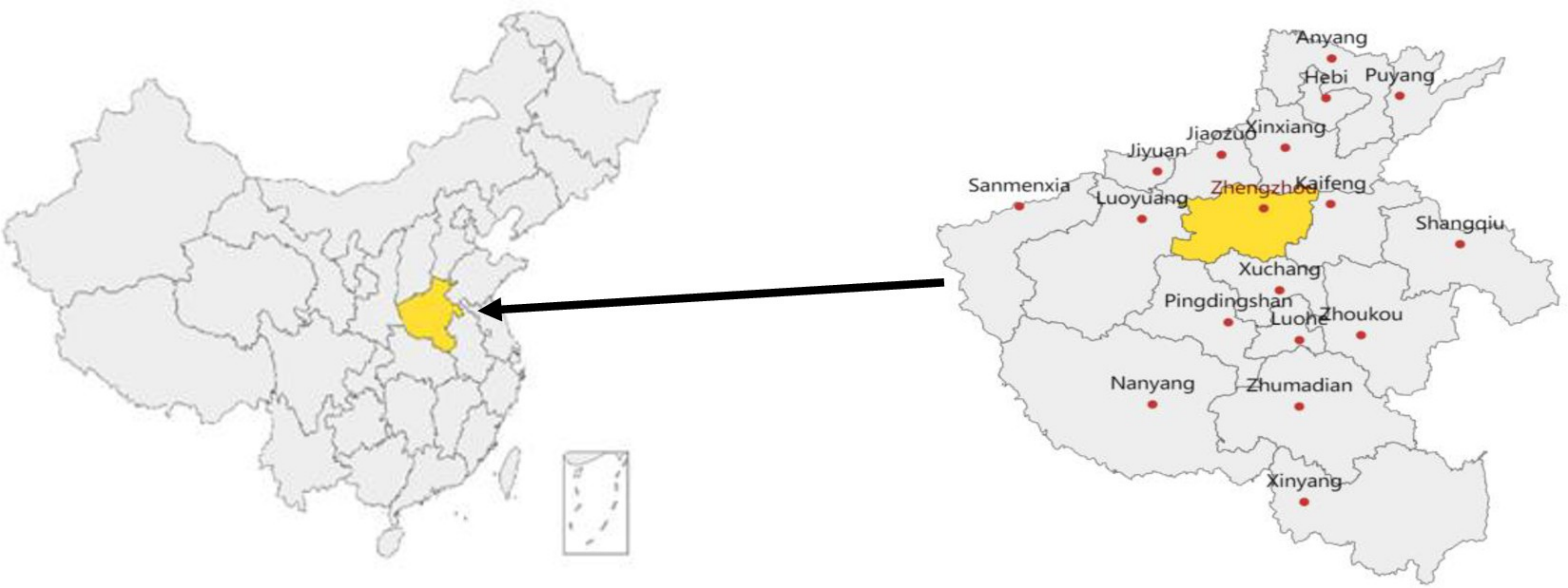
The location of Henan in China and 18 cities in Henan. Note: The map is drawn by the author through Python module, namely pyecharts. Please refer to https://05x-docs.pyecharts.org/#/.

The location of Henan determines that the social development is closely related to many provinces in China, and it also bears the major national strategic arrangements such as The Belt and Road Initiative. However, compared with the eastern coastal areas, the factors of production of Henan have no obvious advantages, and the complicated historical factors have deepened the poverty level of Henan relative to the whole country. At the same time, Henan is a big province of economy, population and agriculture. Henan’s transportation network extends in all directions. Railways, highways and aviation constitute a convenient three-dimensional transportation system, which is an artery of China’s economic and social development. In addition, Henan is facing the situation of slow economic development, weak industrial foundation and fragile ecological environment. Studying the poverty alleviation efficiency in Henan can reflect the poverty alleviation effect with China characteristics. The location of Henan in China and the distribution of 18 cities in Henan are shown in Fig 1.

#### Financial poverty alleviation efficiency accounting model

Output-oriented DEA-BCC model. Data Envelopment Analysis (DEA) is a nonparametric programming method for evaluating the effectiveness of decision-making units using multi-input and multi-output, which was first proposed by famous operations researchers A Charnesand Cooper [54] in 1978. DEA includes CCR model with constant return to scale and BCC model with variable return to scale. This study chooses BCC model to measure the poverty alleviation efficiency of each decision-making unit.

Supposing that there are *n* DMUs to be evaluated, and each DMU has *m* input-variables and *s* output-variables, whose input- and output matrices are *x*_*j*_ = (*x*_1*j*_, *x*_2*j*_,…,*x*_*mj*_)^*T*^ and *y*_*j*_ = (*y*_1*j*_, *y*_2*j*_,…,*y*_*sj*_)^*T*^, and corresponding weight matrix are *v* = (*v*_1_, *v*_2_,…,*v*_*m*_)^*T*^ and *u* = (*u*_1_, *u*_2_,…,*u*_*s*_)^*T*^ respectively, the evaluation equation of each decision-making unit can be obtained as Formula (1):

$$\eta_{j}=\frac{u^{T}y_{j}}{v^{T}x_{j}}=\frac{\sum_{r=1}^{s}u_{r}y_{rj}}{\sum_{i=1}^{m}v_{i}x_{ij}},j=1,2,\cdots ,n$$ (1)

and output-oriented DEA-BCC model can be get as Formula (2):

$$min\sum_{j=1}^{n}v_{j}\cdot x_{j0}-w$$

Subject to:

$$\sum_{i=1}^{m}u_{i}\cdot y_{i0}=1$$ (2)

$$\sum_{i=1}^{m}u_{i}\cdot y_{ik}-\sum_{j=1}^{n}v_{j}\cdot x_{jk}+w\leq 0,\;\mathrm{for}\;k=1,2,\ldots ,h$$

Where, *n* signifies the number of inputs analyzed; *h* represents the number of *DMUs* analyzed; *m* denotesthe number of outputs analyzed; *x*_*jk*_ signifies the amount of input *j* of *DMU k*; *y*_*ik*_ signifies the amount of output *i* of *DMU k*; *x*_*j*0_ denotes the amount of input *j* of the *DMU* under analysis; *y*_*i*0_ signifies the amount of output *i* of the *DMU* under analysis; *v*_*j*_ denotes the weight of input *j* for the *DMU* under analysis; *u*_*i*_ signifies the weight of output *i* for the *DMU* under analysis; *w* denotes the weight coefficient, without restriction of signal.

#### Indicator selection

The above DEA financial poverty alleviation performance evaluation model is affected by various factors, which are difficult to quantify. Comprehensively considering the feasibility of index selection, as well as the availability of data, this study, drawing on existing literature and from the perspective of financial poverty alleviation hardware and software input, selects four indicators namely the number of bank outlets per 10^4^ person etc. to reflect financial poverty alleviation inputs, and selects the per capita disposable income of rural residents as the output of financial poverty alleviation efficiency. Table 1 lists the selected input-output variables (Number of bank branches per 10^4^ people (*Nb*), per capita farm-related loans of rural population (*Lp*), number of employees (*Lb*), Balance of pro-poor micro-credit (*Bp*), and Per capita disposable income of rural residents (*Dr*)).

**Table 1:** Variables selected to assess the efficiency of financial poverty alleviation.

Variable classification	Variable symbol	Unit	Description
Input variables	Number of bank branches per 10^4^ people (*Nb*)	/	Number of bank branches / total resident population (10^4^ person)
	per capita farm-related loans of rural population (*Lp*)	/	Agricultural-related loan balance / agricultural population
	number of employees (*Lb*)	10^4^ person	number of employees
	Balance of pro-poor micro-credit (*Bp*)	10^4^ Yuan	Balance of pro-poor micro-credit
output variable	Per capita disposable income of rural residents (*Dr*)	10^4^ Yuan	Per capita disposable income of rural residents

*Nb* and *Lb* are hardware inputs, and *Nb* indicates the accessibility of financial services. *Bp* and *Dr* are software inputs, reflecting the direct investment in financial poverty alleviation.

#### Econometric model of influencing factors

*Panel Tobit model*. In the process of influencing factor analysis, because the efficiency value calculated by DEA method is selected as the explained variable, the value is 0 and 1. If the least square method is selected for regression, the parameter estimation will be biased and inconsistent. Therefore, in order to better analyze the factors affecting efficiency, this paper selects Tobit model for regression analysis. Tobit model, also known as censored regression model, refers to a class of models in which dependent variables are approximately continuously distributed on positive values, but contain a part of the observed values with positive probabilities of 0. The form of the equation is as shown in Formula (3):

In the process of influencing factor analysis, since the efficiency value calculated by DEA method is chosen as the explanatory variable, there are cases where the value takes 0 or 1. If the ordinary least squares method is chosen for regression, the parameter estimates will be biased and inconsistent. Therefore, to better analyze the influencing factors of efficiency, the Tobit model is chosen for regression analysis. Tobit model, also known as censored regression model, is a class of models in which the dependent variable, although roughly continuously distributed in positive values, contains a fraction of observations that take on the value of zero with positive probability. The specification is shown in Eq (3).

$$y_{i}^{*}=x_{i}\beta +\varepsilon_{i}$$

$$y_{i}=\left\{ \begin{array}{l} 0,\;y_{i}^{*}\leq 0\\ y_{i}^{*},\;y_{i}^{*}>0 \end{array}\right.$$ (3)

Where:

$y_{i}^{*}$ is the unobserved (“latent”) variable; *y*_*i*_ is the observed variable; *x*_*i*_ are explanatory variables; *β* are the unknown parameters; *ε*_*i*_ is normally distributed drawn from *N*(0, *σ*^2^).

To enhance the certainty of the model regression results, this study uses the bootstrap method, proposed by Efron [55], to resample the original sample and standard errors. This sampling method resamples the observed information and thus makes statistical inferences about the overall distributional properties, which can improve the accuracy and standard errors of the prediction model. In this study, the nonparametric bootstrap method was employed, and the sample size selected is 999.

In addition, Tobin panel Tobit regression model [56] is selected to estimate the overall sample, and the distribution function is presented in Eq (3). According to the model (4), when the financial poverty alleviation efficiency (*Ef*_*it*_ = 1) in year *t* of the *i*th region is 1, it will be compressed into a point. At this time, the probability distribution of *Ef*_*it*_ will become a mixed distribution, consisting of a discrete distribution (the case of *Ef*_*it*_ = 1) and a continuous distribution (the case of *Ef*_*it*_<1). Therefore, this study adopts the panel Tobit model with bootstrap method, which takes into account not only the distribution of the dependent variable as a mixed distribution, but also the deterministic issue of the prediction model.

#### Indicator selection

Dependent variable. (1) Efficiency of financial poverty alleviation (*Ef*_*it*_). Efficiency refers to the evaluation method of using resources most effectively to meet the given wishes and needs under the given conditions of input and technology. DEA is a linear programming model, expressed as a ratio of outputs to inputs. By comparing the efficiency of a specific decision-making unit with the performance of a group of decision-making units that provide similar services, the efficiency of the specific decision-making unit is maximized. In this process, the units that obtain 100% efficiency are referred to as relatively efficient units, while the other units with efficiency scores below 100% are referred to as inefficient units. In this study, we use the Output-oriented DEA method to construct an efficiency accounting model with four inputs and one output. The specific indicators are shown in Table 2.

**Table 2:** Variables and their descriptions.

Variable classification	Variable symbol	Description
Dependent variable	Efficiency of financial poverty alleviation (*Ef*_*it*_)	Measured efficiency of poverty alleviation in each city
Independent variable	Dualistic economic structure (*De*_*it*_)	Urban per capita disposable income/rural per capita net income
	Industry structure (*Is*_*it*_)	Total output value of primary and tertiary industries/GDP
	Agriculture-related financial subsidies (*Fs*_*it*_)	Amount of financial support for agriculture/total regional rural population
Control variable	Overall level of financial development (*Ol*_*it*_)	Regional institutional loan balance / Regional GDP
	Per capita beneficial agriculture points (*Ba*_*it*_)	Total number of regional beneficiary payment points/regional population
	The extent of financial benefits to farmers (*Eb*_*it*_)	Balance of agricultural-related loans from financial institutions / GDP of primary industry

*Independent variables*. (1)Dualistic economic structure(*De*_*it*_). China has a unique urban-rural dualistic economic structure, which has a profound impact on the migration and allocation of financial capital between urban and rural areas. As China’s reform enters a deeper and more difficult period, the lack of regulation in the financial market has led to a lack of financial incentives to help the poor among Chinese regions, which is increasingly reflected through the dualistic economic structure. The urban-rural income ratio, i.e. urban per capita disposable income / rural per capita net income, is used to examine the impact of the dualistic economic structure on financial poverty alleviation.

(2) Industry structure (*Is*_*it*_). Henan is located in the plain area with developed agriculture, abundant grain crops. The arable land area of Henan is 6.871 million hectares, ranking second in China. It is China’s major province of grain production and labor export. At the same time, Henan is the birthplace of Chinese civilization, with abundant natural and cultural tourism resources. Therefore, an important way to help the poor through finance in Henan is to adjust the industrial structure according to the unique natural and cultural resources, develop characteristic ecological agriculture and tourism, and transform the green mountains and rivers and precious folk culture into the driving force to eliminate poverty. Hence, industry structure, namely the proportion of gross output value of primary industry and tertiary industry to GDP, is selected to measure the impact of industrial structure on the efficiency of financial poverty alleviation.

(3) Agriculture-related financial subsidies (*Fs*_*it*_). Poverty alleviation is a systematic project, and financial poverty alleviation requires the cooperation of all parties, especially the investment of government in the construction of financial ecology, including rural infrastructure construction, rural financial publicity and education bases, and government financial subsidies for poverty alleviation credit. Agriculture-related financial subsidies are conducive to improving rural infrastructure construction, laying the foundation for the development and growth of local characteristic advantageous industries, providing an effective carrier for financial poverty alleviation, and also alleviating the rejection of financial institutions for poverty alleviation in remote rural poor areas. In addition, sufficient financial subsidies can ensure that financial capital can participate in poverty alleviation without worries and stimulate financial institutions to invest financial capital in poor rural areas. Therefore, the financial subsidies, namely per capita amount of agriculture-related financial subsidies, is chosen to examine the impact of financial subsidies on the efficiency of financial poverty alleviation.

*Control variables*. (4) Overall level of financial development (*Ol*_*it*_). The overall level of financial development is the basis for financial poverty alleviation in a region. The overall level of financial development, i.e., regional institutional loan balance / regional GDP, is chosen to examine the impact of the overall level of financial development in different regions on the efficiency of financial poverty alleviation.

(5) Per capita payment services points for farmers (*Ps*_*it*_). The payment services for farmers refers to the basic payment and settlement services such as small withdrawal, balance inquiry, cash remittance, transfer and remittance as well as agent payment provided by bank card acquirers to debit card holders by deploying bank card acceptance terminals at designated cooperative merchant service points in rural villages and townships.

The payment services points for farmers is an extension point for further implementing the policy of strengthening farmers and benefiting farmers, effectively improving the payment environment in rural areas, and providing farmers with low-cost, convenient and fast financial payment services. It is selected to examine the impact of the heterogeneous characteristics of per capita payment services points for farmers on the efficiency level of financial poverty alleviation in different regions.

(6) The extent of financial benefits to farmers (*Eb*_*it*_). The difference in the degree of financial support for farmers directly affects the availability and cost of financial services in poor rural areas, which in turn has a bearing on whether poor farmers can obtain sufficient funds to carry out production to get out of poverty and become rich. The extent of financial benefits to farmers, i.e., the ratio of balance of financial institutions’ agricultural-related loans to GDP of primary industry, is chosen to examine the impact of different degrees of financial support on the efficiency level of financial poverty alleviation performance in different regions.

Based on the above variable selection and Tobit model, the following econometric model (4) was constructed:

$${Ef}_{it}=\left\{ \begin{array}{l} \;\alpha_{0}+\alpha_{1}{De}_{it}+\alpha_{2}{Is}_{it}+\alpha_{3}{Fs}_{it}+\sum_{n=1}^{3}\beta_{n}{con}_{it}+\varepsilon_{it},\;{Ef}_{it}<1\\ 0,\;{Ef}_{it}=1 \end{array}\right.$$ (4)

Where *con*_*it*_ is the three control variables considered in this study (Overall level of financial development, Per capita beneficial agriculture points, and The extent of financial benefits to farmers). *α*_0_ is the constant term. *α*_1_, *α*_2_, *α*_3_, and *β*_*n*_ are the coefficients on each variable. *ε*_*it*_ is the error term. Table 2 lists all the variables and their definitions.

### Data source

Henan is China’s largest agricultural province, as well as a province with a large agricultural population and a huge rural poor population. The poverty-stricken areas and poor population in Henan have weak self-development ability and high rate of return to poverty. Most of the poor people are concentrated in the traditional plain agricultural areas, the Yellow River floodplains, the mountainous areas of western Henan and the old revolutionary areas. These areas are far away from urban centers and main traffic lines, with poor geographical location and natural resource conditions, coupled with the poor education level of the poor, making them over-reliant on agriculture, especially planting.

In addition, due to the low degree of commercialization and marketization of agricultural products in these regions, the regionalization trend of poverty is obvious. To carry out targeted poverty alleviation work, the Chinese government has designated 832 national-level poverty-stricken counties on a county-by-county basis based on indicators such as the average annual net income of local residents. As of October 2019, there are 485 national-level poverty-stricken counties in China, 14 of which are in Henan. Among them, Nanyang City accounted for 4 poor counties, 3 in Zhumadian City, 2 in Luoyang City and Puyang City each, and 1 in Pingdingshan City, Sanmenxia City and Xinyang City each.

The data in this study come from China Social Statistical Yearbook, Henan Statistical Yearbook, Statistical Bulletin of National Economic and Social Development of Henan, China Financial Statistical Yearbook, Statistical Bulletin of National Economic and Social Development of each city in Henan, website of China Banking Regulatory Commission, Zhengzhou Central Branch of People’s Bank of China, etc. DEA analysis was conducted using Deap2.1 software, and the data were standardized before analysis. Tobit analysis was performed using State software. Descriptive statistics of the data are shown in Table 3.

**Table 3:** Descriptive statistics of the variables.

Variables		SD	max	min	Mean
DEA	*Nb*	1.711	-0.335	-1.814	-0.566
	*Lp*	1.508	-0.110	-1.252	-0.679
	*Lb*	7.041	11.608	8.625	8.750
	*Bp*	7.203	11.983	8.116	8.965
	*Dr*	13.388	10.666	10.270	10.456
Tobit	*Ef*_*it*_	1.899	0.000	-0.503	-0.054
	*De*_*it*_	0.772	1.023	0.449	0.618
	*Is*_*it*_	2.135	-0.352	-0.576	-0.509
	*Fs*_*it*_	1.674	6.911	5.323	6.477
	*Ol*_*it*_	9.361	8.080	7.605	7.938
	*Ba*_*it*_	0.641	-0.689	-1.650	-0.717
	*Eb*_*it*_	5.338	6.911	6.227	6.645
	*Gdp*_*it*_	11.524	11.447	10.979	11.158

Note: The above data are from publicly published data sources and the author’s calculations. A logarithmic operation is performed on the data. The original dataset can be accessed upon request.

## Results and discussion

### Results of financial poverty alleviation efficiency measurement and discussion

According to the method and selected indicators provided in Section 3.1, the financial poverty alleviation efficiency of 18 regions in Henan from 2009–2018 was accounted for using DEAP2.1 software, and the results are shown in Table 4. Based on the above DEA measurement results, the financial poverty alleviation efficiency of 18 regions in Henan from 2009–2018 can be divided into three groups: 2 cities in the excellent group, including Zhengzhou and Luoyang; 8 cities in the modest group, including Xuchang, Sanmenxia, Jiyuan, Jiaozuo, Xinxiang, Hebi, Puyang, and Anyang; and the 8 remaining cities in the poor group.

**Table 4:** Assessment results of financial poverty alleviation efficiency in various regions of Henan.

	2009	2010	2011	2012	2013	2014	2015	2016	2017	2018	Mean
**zhengzhou**	1.000	1.000	1.000	1.000	1.000	1.000	1.000	1.000	1.000	1.000	1.000
**luohe**	0.781	0.787	0.789	0.795	0.794	0.798	0.805	0.803	0.811	0.814	0.798
**xuchang**	0.875	0.883	0.891	0.897	0.904	0.915	0.919	0.938	0.952	0.969	0.914
**pingdingshan**	0.867	0.875	0.870	0.874	0.879	0.882	0.889	0.894	0.895	0.898	0.882
**Mean**	0.881	0.886	0.888	0.892	0.894	0.899	0.903	0.909	0.915	0.920	0.899
**zhoukou**	0.641	0.645	0.649	0.655	0.662	0.664	0.665	0.667	0.669	0.668	0.659
**shangqiu**	0.795	0.807	0.823	0.832	0.845	0.855	0.865	0.885	0.891	0.895	0.849
**kaifeng**	0.761	0.764	0.770	0.773	0.781	0.793	0.834	0.847	0.879	0.908	0.811
**Mean**	0.732	0.739	0.747	0.753	0.763	0.771	0.788	0.800	0.813	0.824	0.773
**nanyang**	0.641	0.651	0.659	0.663	0.679	0.702	0.714	0.724	0.743	0.768	0.694
**xinyang**	0.677	0.684	0.687	0.690	0.694	0.699	0.704	0.710	0.715	0.713	0.697
**zhumadian**	0.605	0.612	0.622	0.627	0.634	0.650	0.659	0.669	0.675	0.680	0.643
**Mean**	0.641	0.649	0.656	0.660	0.669	0.684	0.692	0.701	0.711	0.720	0.678
**luoyang**	1.000	1.000	1.000	1.000	1.000	1.000	1.000	1.000	1.000	1.000	1.000
**sanmenxia**	0.935	0.937	0.940	0.945	0.943	0.944	0.945	0.950	0.953	0.952	0.944
**Mean**	0.968	0.969	0.970	0.973	0.972	0.972	0.973	0.975	0.977	0.976	0.972
**jiyuan**	0.945	0.959	0.969	0.994	0.932	0.941	0.964	0.968	1.000	1.000	0.967
**jiaozuo**	0.914	0.919	0.924	0.927	0.932	0.930	0.937	0.943	0.948	0.954	0.933
**xinxiang**	0.929	0.938	0.948	0.946	0.957	0.963	0.965	0.967	0.972	0.981	0.957
**hebi**	0.978	0.982	0.986	0.992	0.899	0.900	0.907	0.909	0.908	0.911	0.937
**puyang**	0.931	0.937	0.942	0.950	0.962	0.967	0.973	0.981	0.984	0.987	0.961
**anyang**	0.894	0.896	0.902	0.912	0.920	0.926	0.938	0.947	0.959	0.969	0.926
**Mean**	0.932	0.939	0.945	0.954	0.934	0.938	0.947	0.953	0.962	0.967	0.947

Data source: Author’s calculations.

From the results shown in Table 4, the financial poverty alleviation in Henan from 2009 to 2018 is relatively efficient. The efficiency values of financial poverty alleviation in Zhengzhou City and Luoyang City are both 1, which indicates that the development scale of financial poverty alleviation in these two cities is reasonable and resources have been effectively utilized to maximize the effect of poverty alleviation. The overall efficiency of financial poverty alleviation in Jiyuan City has increased over years. it rose to 1 in 2017 and maintained until 2018, indicating that Jiyuan City has made greater efficiency progress in the financial poverty alleviation. Xuchang, Sanmenxia, Jiaozuo, Xinxiang, Hebi, Puyang, and Anyang have seen their financial poverty alleviation rise yearly, and their values have approached 1, i.e., they are close to being efficient. Zhoukou, Xinyang, and Zhumadian cities have lower comprehensive efficiency values, indicating that there is much room for improving the efficiency of financial poverty alleviation, and they can continue to increase the investment of financial capital to improve poverty alleviation efficiency.

In addition, from the perspective of the time sequence evolution of financial poverty alleviation efficiency in 18 regions from 2009 to 2018, the financial poverty alleviation efficiency of each region in Henan has a relatively similar trend of change. The efficiency in Zhengzhou and Luoyang is relatively stable, maintaining an efficiency value level of 1 during the sample period, so the two cities can be called stable efficiency cities. The efficiency values of Jiyuan and Hebi cities fluctuated, so the two cities can be called fluctuating efficiency cities. Other cities in the sample period have steadily increased their efficiency values, which can be called stable and improving efficiency cities.

In summary, the financial poverty alleviation efficiency in the 18 sample cities from 2009 to 2018 showed a trend of diminishing returns to scale except for Zhengzhou and Luoyang. In terms of input redundancy, the outlets of the financial institution were relatively over-invested but did not obtain the desired effect, indicating that the financial poverty alleviation work in the sample period focused more on the quantitative expansion of rural financial institutions and less on the internal management, technological innovation, rational allocation of financial resources and improvement of financial service quality. Future financial poverty alleviation policies should regulate this situation.

### Determinants of financial efficiency in poverty alleviation and discussion

This section introduces the results of Tobie regression according to the selected indicators and constructed models in Section 3.2.

Pseudo regressions may be caused by sequence instability. To avoid spurious regressions, this study performed unit root tests for selected variables using two methods, namely Harris-Tzavalis [57] (HT-Test) and Fisher-ADF test (enhanced Dickey-Fuller) [58]. The HT test allows the use of common roots across regions, while the Fisher-ADF test inspects a single root for each city under the null hypothesis that all panels contain unit roots [59].

The results of the unit root tests for each variable are presented in Table 5. the HT test results indicate that there are no cases where the variables contain a common unit root among all panel data variables. The heterogeneous Fisher-ADF unit root test for all variables rejects the null hypothesis at the 10% significance level. The results of the combined two unit root tests indicate that all panel data variables are stable, laying the groundwork for subsequent regression estimation.

**Table 5:** Panel unit root results for selected variables.

Variables	HP	Fisher-ADF
*Ef*_*it*_	0.2152*** (0.0001)	4.5639*** (0.0007)
*De*_*it*_	0.3822*** (0.0000)	5.3078*** (0.0000)
*Is*_*it*_	0.4027*** (0.0000)	6.5192** (0.0000)
*Fs*_*it*_	0.3571*** (0.0000)	2.5943** (0.0451)
*Ol*_*it*_	0.3957***(0.0003)	3.5281*** (0.0000)
*Ps*_*it*_	−0.0379*** (0.0000)	1.3029*** (0.0000)
*Eb*_*it*_	0.5001*** (0.0005)	4.3584*** (0.0000)

Notes: The figures in brackets denote the z-statistics of the coefficients. Upper corner markers, ^⁎⁎⁎^, ^⁎⁎^, and ^⁎^ indicate rejection of the null at 1%, 5%, and 10%, respectively.

With annual data for 18 cities from 2009–2018, a panel-Tobit model with bootstrap regression was employed, and the results are given in Table 6. Models (A), (B), and (C) use three core variables, i.e., Dualistic economic structure (*De*_*it*_), Industry structure (*Is*_*it*_), and Agriculture-related financial subsidies (*Fs*_*it*_). Model (D) contains three core variables at the same time. Model (E) considers three control variables, i.e., Overall level of financial development (*Ol*_*it*_), Per capita Payment service points for farmers (*Ba*_*it*_), The extent of financial benefits to farmers (*Eb*_*it*_), based on model (D). The following conclusions can be drawn from the regression results.

**Table 6:** Results of Tobit model with bootstrap regression for the financial poverty alleviation efficiency.

Variables	(A)	(B)	(C)	(D)	(E)
*De*_*it*_	-0.1023***(1.20)			-0.8054 ***(4.12)	-0.1106***(4.01)
*Is*_*it*_		0.0851***(-5.37)		0.0797***(2.19)	0.0716**(3.92)
*Fs*_*it*_			0.2016*** (0.51)	0.1934 **(4.11)	0.2133***(3.32)
*Ol*_*it*_					0.0039**(2.27)
*Ps*_*it*_					0.0018**(1.31)
*Eb*_*it*_					0.0031*(3.58)
*_cons*	-0.159***(-10.12)	-1.159***(-6.12)	0.217***(2.13)	1.695***(3.86)	0.2170***(4.02)
*Sigma_u*	0.842***(11.37)	0.958***(16.25)	1.009***(9.82)	1.329***(9.82)	1.122***(7.97)
*Sigma_e*	0.468***(9.62)	0.376***(11.30)	3.95***(7.51)	3.950***(6.03)	2.837***(2.11)
*N*	18	18	18	18	18

(1) Dualistic economic structure is one of the determinants of the efficiency of financial poverty alleviation. Urban-rural dualistic economy structure refers to the widely existing asymmetry of urban-rural production and organization in developing countries, and also a socio-economic form in which the backward traditional agricultural sector and modern economy coexist with obvious disparity. The urban-rural dualistic economic structure was gradually formed with the emergence of cities. The process of urban emergence is the process of urban-rural separation and antagonism. The separation of urban and rural areas and the formation of urban-rural dualistic economic structure on this basis is mainly formed by the difference in productivity development between urban and rural areas, and becomes increasingly prominent as this difference expands. Development economics believes that the process of economic development is a process of continuous transformation of economic and social structures.

The regression results show that the urban-rural dualistic economy structure is negatively related to financial poverty alleviation performance. More specifically, for every 1% increase in the value of the urban-rural dualistic economy structure, the efficiency of poverty alleviation decreases by 0.11%. The dualistic economic structure leads to a dualistic financial structure, and China is a typical developing country with a dualistic economic structure and a dualistic financial structure. The developed economy and abundant investment and employment opportunities in cities attract a large influx of capital and talent, which also includes a large outflow of highly educated talent from rural areas. This has resulted in a lack of resources for development in rural areas, which is a serious impediment to economic growth and poverty alleviation in rural areas.

(2) Industrial structure is a determinant of the efficiency of financial poverty alleviation. As shown in model (E) of Table 6, industrial structure has a positive effect on financial poverty alleviation efficiency with a 5% significance.

More specifically, for every 1% increase in the value of the industrial structure, the efficiency of poverty alleviation will rise by 0.072%, thus enhancing the effectiveness of financial poverty alleviation. For farmers, agriculture is the basis for effectively improving their economic income, and the organic combination of agriculture and tertiary industry is the effective way to improve their poverty status. When carrying out industrial poverty alleviation, financial institutions in each region should combine special agricultural products and green agricultural products with special tertiary industries (such as specialty tourism and specialty catering). Providing employment opportunities for poor farmers through special agriculture and expanding the marketing and sales of agricultural products through special service industry to truly realize the growth of farmers’ income and help poor farmers achieve poverty alleviation.

It is worth noting that increasing the proportion of primary industry or reducing the proportion of tertiary industry alone will not necessarily improve the efficiency of the financial sector in poverty alleviation. Simply increasing the share of the primary sector without reliable consumption channels may lead to overcapacity in the primary sector, which will definitely lower the price of agricultural products and may result in the phenomenon of low grain prices hurting farmers. The general consumption of agricultural products is protected by the state price, so it is difficult to create a driving force in poverty alleviation effect. Only specialty agriculture can stimulate consumption desire through the tertiary industry, increase farmers’ income, and realize the improvement of poverty alleviation efficiency.

(3) Financial subsidies are the most important affecting factor of financial poverty alleviation efficiency. Model (E) shows that financial subsidies are positively associated with financial poverty alleviation at the 1% level of significance. More specifically, for every 1% increase in the value of financial subsidies, the efficiency of poverty alleviation increases by 0.213%. For poor areas, they do not have the advantage of economic development, coupled with the concentration of poor people, weak industrial strength, and poor infrastructure construction, it is necessary to give full play to the macro-control functions of the government to achieve poverty alleviation. The government’s financial subsidies can play a blood-making function, which helps to explore the advantages of regional resources, adjust the industrial structure and improve the level of infrastructure construction, thus revitalizing the market of poor regions.

In addition, financial support for poverty alleviation has a siphon effect, which attracts a large amount of private capital to the poverty alleviation field in addition to the poverty alleviation effect of the financial subsidies themselves. Poverty eradication is a national strategy in China, and the Chinese government has always regarded poverty reduction as an important goal of national development. Since the 1980s, the Chinese government has been carrying out rural poverty alleviation in an organized, planned and large-scale manner, and has formulated and implemented many poverty reduction plans, making poverty alleviation the consensus and action of the whole China. The shortage of funds is an important factor limiting the survival and development of the poor, for which the Chinese government has invested in multiple types and large amounts of subsidies. For example, from 2016 to 2020, the Chinese government has added 20 billion yuan of special central financial funds for poverty alleviation each year for five consecutive years, reaching 146.1 billion yuan in 2020, playing the role of the main channel for precise poverty alleviation funds.

(4) The regression results of the control variables show that three control variables, namely, Overall level of financial development (*Ol*_*it*_), Per capita beneficial agriculture points (*Ba*_*it*_), and The extent of financial benefits to farmers (*Eb*_*it*_) affect the efficiency of financial poverty alleviation at 5%, 5%, and 10% significance levels, respectively, and the regression results are as expected. A higher overall level of financial development (*Ol*_*it*_) does not only represent a more prosperous economy, but also contains more resources such as talents and equipment to invest in economic development. The impact of per capita beneficial agriculture points (*Ba*_*it*_) on the efficiency of financial poverty alleviation is complex, but currently Henan is still at the stage where increasing per capita beneficial agriculture points (*Ba*_*it*_) can improve the efficiency of financial poverty alleviation. However, the number of beneficial agriculture points should not be blindly increased, otherwise it may produce negative effects. It is necessary to vigorously increase the extent of financial benefits to farmers(*Eb*_*it*_), which will not only improve the efficiency of financial poverty alleviation, but also enable the majority of poor farmers to obtain well-being.

### Robustness test

The robustness test examines of the interpretation ability of evaluation methods and indicators, that is, whether the evaluation methods and indicators still maintain a consistent and stable explanation of the evaluation results when some parameters are changed [15]. The commonly used method of robustness test include variable replacement, change sample volume, sub-sample regression, supplementary variable and so on. The method to be selected for robustness testing varies with the purpose of the study. According to the research purpose, the following two robustness tests are carried out on the model (E) in Table 6 in this study. The first one is changing the estimation method, i.e., re-estimating the parameters using the panel Tobit model of maximum likelihood SE. The second one is the supplementary variable method, i.e., adding a dummy variable, namely the regional economic development level, in the regression; it should be noted that the regression method still adopts the Panel-Tobit model with bootstrap regression. The results are shown in Table 7.

**Table 7:** Results of Tobit model of maximum likelihood SE for different regions.

	*Coef*.	*z*	*p-value*
*De*_*it*_	-0.1011	4.16	0.000***
*Is*_*it*_	0.0671	2.17	0.017**
*Fs*_*it*_	0.2846	3.05	0.000***
*Ol*_*it*_	0.0037	2.33	0.006***
*Ps*_*it*_	0.0019	1.50	0.025*
*Eb*_*it*_	0.0023	2.89	0.037*
*_cons*	0.2130	7.03	0.000***
*Sigma_u*	1.2066	2.11	0.000***
*Sigma_e*	2.5063	2.07	0.000***

Notes

^⁎⁎⁎^, ^⁎⁎^, and ^⁎^ indicate rejection of the null at 1%, 5%, and 10%, respectively.

Table 7 shows the regression results of the Panel-Tobit model with maximum likelihood SE, and Table 8 shows the regression results considering the dummy variable of regional economic development. According to Tables 7 and 8, although some variations are reported in the significance level of the coefficients of individual control variable, their values, symbols and significance levels do not change significantly. Therefore, the regression results in this study are relatively robust.

**Table 8:** Results of robustness test on joining GDP dummy variable for different regions.

	*Coef*.	*z*	*p-value*
*De*_*it*_	-0.1037	3.29	0.000***
*Is*_*it*_	0.0695	3.27	0.013**
*Fs*_*it*_	0.2652	2.99	0.000***
*Ol*_*it*_	0.0048	2.86	0.017**
*Ps*_*it*_	0.0023	1.33	0.018****
*Eb*_*it*_	0.0027	3.62	0.011**
*Gdp*_*it*_	0.9523	3.92	0.020**
*_cons*	0.2099	7.65	0.000***
*Sigma_u*	1.1520	3.15	0.000***
*Sigma_e*	2.9201	2.13	0.000***

Notes

^⁎⁎⁎^, ^⁎⁎^, and ^⁎^ indicate rejection of the null at 1%, 5%, and 10%, respectively.

## Conclusions and policy recommendations

### Conclusions

This study adopts the DEA method to evaluate the efficiency of financial poverty alleviation in different regions based on the data of 2009–2018 of Henan, a major agricultural province of China. In addition, using the Panel-Tobit model with bootstrap regression, this study explores the influence mechanism of financial poverty alleviation efficiency from the perspectives of dualistic economic structure, industrial structure and financial subsidy, identifing the key determinants of financial poverty alleviation efficiency in different regions. The empirical results are indicative of the following conclusions:

First, during the sample period, the efficiency of financial poverty alleviation in Henan has an obvious upward trend, which is the result of the rapid development of China’s social financial industry and the government’s high emphasis on poverty alleviation. In recent years, the Chinese government has continuously highlighted the importance of poverty alleviation, invested a large amount of resources, innovated institutional mechanisms, and implemented the inclusive financial system, gradually formed a scalable and sustainable financial poverty alleviation model with Chinese characteristics.

Secondly, there were significant heterogeneities in the efficiency of financial poverty alleviation in different regions. The efficiency values of financial poverty alleviation in Zhengzhou and Luoyang reached 1, indicating that the financial poverty alleviation in the two regions were efficient and achieved Pareto optimality; The financial poverty alleviation projects in the remaining 16 regions still had different degrees of room for progress in financial poverty alleviation; The financial poverty alleviation project in Jiyuan has made remarkable progress; Xuchang, Sanmenxia, Jiaozuo, Xinxiang, Hebi, Puyang and Anyang have made steady progress in the efficiency of financial poverty alleviation; The efficiency of financial poverty alleviation in Zhoukou, Xinyang and Zhumadian was low and needed to be improved. The efficiency of financial poverty alleviation in Zhengzhou and Luoyang was relatively stable, which could be called stable efficiency cities. The efficiency values of Jiyuan and Hebi fluctuated, which could be called fluctuating efficiency cities. The efficiency of financial poverty alleviation in other cities has increased steadily, which can be called efficiency stable cities. It is worth noting that the financial poverty alleviation efficiency of the sample cities in the sample period, except Zhengzhou and Luoyang, showed a decreasing trend of return to scale; Improving the allocation of input resources and thus the efficiency of financial poverty alleviation has become an important way to improve the efficiency of financial poverty alleviation in the future.

Thirdly, in terms of the factors affecting the efficiency of financial poverty alleviation, the dualistic economy structure, industrial structure and financial subsidies were all important factors influencing the efficiency of financial poverty alleviation. Among them, the influence of financial subsidies was the most significant, followed by the dualistic economy structure and finally the industrial structure. In addition, financial subsidies can leverage more resources to flow to rural poor areas, which plays a decisive role in financial poverty alleviation in Henan. It is worth noting that the dualistic economic structure between urban and rural areas has a negative impact on the efficiency of financial poverty alleviation, which is universal for developing countries. The improvement of industrial structure and financial subsidies can effectively alleviate the main economic contradictions in poor areas, which is conducive to enhancing the export of products from poor areas and raising the income level of poor people.

### Policy recommendations

Based on the findings of this study, the following policy recommendations are proposed to enhance the efficiency of financial poverty alleviation in Henan:

Firstly, in view of the significant heterogeneity of financial poverty alleviation efficiency in different regions, a precise mechanism for financial poverty alleviation should be established, e.g. implement differentiated subsidies for different regions and increase financial subsidies for areas with low financial poverty alleviation efficiency. For some deep and contiguous poverty-stricken areas with large poverty population, high incidence of poverty, and great difficulty in getting rid of poverty, it is neither realistic nor effective to adjust the industrial structure and arrange more financial outlets in a short period of time. In these cases, more financial subsidies can be used to improve their financial poverty alleviation efficiency. On the basis of financial subsidies from the central government, cities should also increase their investment in deep poverty areas themselvse to prevent crowding-out effect. Meanwhile, it is necessary to coordinate and integrate the use of agricultural financial subsidies, and closely link the use of funds to poverty alleviation. Implement the requirements of budget performance management, make full use of the dynamic monitoring platform of financial poverty alleviation funds, keep track of the usage of special financial poverty alleviation funds timely, earnestly strengthen the supervision of funds, ensure the quality of poverty alleviation, and establish a long-term mechanism for stable poverty alleviation.

Secondly, judging from the basic situation of Henan’s financial poverty alleviation efficiency and its influencing factors, the first is to continue to promote the reform of the dualistic financial system, break down the barriers of the dualistic economy, and attract more talents and enterprises to the countryside. According to the poverty causes and the difference in credit demand of the rural poor, constantly innovate financial poverty alleviation products and service models, and customize financial poverty alleviation measures. The second is to improve the rural industrial structure. As Henan is located in the regional center of the Central Plains, the birthplace of Chinese civilization and rich in historical and cultural resources, it can support the development of local characteristic industries and advantageous industries according to the differences of resource endowment, historical folk culture and industrial development in poverty-stricken areas.

It is worth noting that although various financial institutions have made a lot of exploration, negative factors such as the lack of effective collateral for rural credit objects and incomplete credit information still exist. Therefore, the local government should rely on financial support to efficiently optimize and strengthen rural industries; Accelerate the development of new agricultural system and improve the rural financial service system; Hasten the innovation of rural financial products and advance the pertinence of financial services; Give full play to the advantages of financial institutions, form a joint force of rural financial work, and promote the development of rural finance.

Thirdly, in addition to the above measures, more measures to improve the efficiency of financial poverty alleviation should be explored according to the influence of the control variables considered in this study. For example, take full advantage of China’s advantages in information infrastructure construction and financial technology, especially under the influence of the epidemic, deeply integrate financial poverty alleviation and Internet Finance, and establish a big data management platform for targeted poverty alleviation, so that limited credit resources and preferential poverty alleviation policies can be reasonably matched with truly poor farmers, optimize the allocation and improve the efficiency of financial poverty alleviation.
